# Steam Gauge

**Published:** 1865-12

**Authors:** 


### STEAM GAUGE.

BY J.

Having read Dr. Lawrence's article in the Register upon the substitution of the Pressure Gauge, for the Thermometer in Vulcanizers, and the greater safety with which they could be used, together with the admirable degree of comfort with which the dentist would use his infernal machine, I concluded to try the gauge, and will give the result of my experiment, for the benefit of your readers. First, let me explain, by saying, I do not, or did not consider the gauge any safer than the thermometer, for the guage certainly adds nothing to the strength of a vulcanizer, and as Dr. Jacksou has written, requires the same degree of attention to obviate the same results of carelessness with a thermometer; consequently, watchfulness is the better safeguard.

It is a well known fact in relation to the pressure of steam, that to be most expansive, or of the most force, it must be well "lubricated," or moistened, and every degree of heat that is added to water, after it has expanded to its utmost, or been crowded into steam, diminishes its expansion, and consequently makes it of less force.

Knowing this fact, I had a hole drilled and tapped in the top of my vulcanizer,—which, by the way is a "Buckeye," for two pieces and attached a steam or pressure guage. With one piece of work which I put into the machine, I also put about one-third of a tumbler of water, certainly not more—fastened on the top, and "fired up," watching both thermometer and guage. They both moved corresponding to the table published by Dr. Lawrence, until the first indicated

320°, and the latter 110 lbs. I then increased the heat to 340°, when the guage commenced to indicate a decreasing pressure, and finally fell back to 30 lbs. At this time I reduced the heat to 320°, this was the relative positions of both, 320° of heat, 30 lbs pressure, for one hour, during which, I continued the experiment, and to me was positive proof of the non-expansion of super-heated steam and the non-reliability of the guage under these circumstances, at least.

My conclusions are that the thermometer is the best guide for us in vulcanizing, and perfectly safe, if the attention which is necessary to a guage is given to it, and certainly the best, if we use but little water; in the latter case much the safest, for we have not steam enough to create a pressure of over 30 lbs., which will not explode any vulcanizer in use. By using very little water, we also save fuel, which is quite an item to those who are paying five dollars a gallon for alcohol.

I might here give a few general rules for handling a vulcanizer, with the best results, and of course it must be understood, that these rules are founded upon our experience. 1st—Use but little water. 2nd—Apply the heat slowly, and consume at least thirty minutes in running the heat up to the vulcanizing point. (300)* 3d—Watch the thermometer attentively for say fifteen minutes, or, until you are satisfied that the heat is uniform, and the mercury will neither rise or descend, and then your apparatus will require little attention until the work is done, which will be one hour and twenty minutes after the mercury has indicated 300° of heat. If these general rules are followed, I think that perfect safety will be the consequence, as I know the best work will be produced.

* As thermometers sometimes vary five or ten degrees, it may be possible that 305° or 310° will be the vulcanizing point. One or two experiments with a tube will be sufficient to satisfy the operator.

